# Stem cell transplantation in aplastic anemia: Impact on choices for first line therapy

**DOI:** 10.1097/HS9.0000000000000249

**Published:** 2019-06-30

**Authors:** Andrea Bacigalupo, Sabrina Giammarco

**Affiliations:** 1 Fondazione Policlinico Universitario A Gemelli IRCCS, Italy; 2 Istituto di Ematologia, Universita’ Cattolica del Sacro Cuore, Roma, Italy

**Take home messages**

- The age effect on survival after allogeneic SCT for AA.
- an age cut off for selecting first line therapy.
- conditioning regimens for matched sibling and unrelated donor transplant.
- the role of prophylactic Rituximab on EBV infections.

## Introduction

The 2 major complications of SCT for acquired aplastic anemia (AA) are graft failure and GvHD. Graft failure remains an issue, more frequent than rejection in patients with acute leukemia. GvHD has no beneficial effect in AA patients, and particular care should be taken to reduce its incidence as much as possible: in vivo T cell depletion with anti-thymocyte globulin (ATG) or alemtuzumab (Campath) is one way to effectively prevent GvHD. We will discuss current status of SCT in acquired AA, and future perspective.

## Current state of the art

### HLA typing of the patient at diagnosis and the age effect

HLA typing of the patient and the family, at the time the patient is diagnosed with aplastic anemia (AA), remains a crucial early diagnostic procedure, and should be considered in every patient up to the age of 60 years. If an HLA identical sibling is available, then SCT is first line treatment in children and in young adults. The question is: what is a young adult. Current guidelines indicate 40 to 50 years as a cut off, because there is a very strong age effect with survival of 86%, 76%, 55% survival at 10 years for patients aged 1 to 20, 21 to 40 and over 40 years,^1^ and survival has not improved in recent years for patients over 40.^2^ Upfront SCT may be carefully considered for selected cases with good performance status and severe disease, aged 40 years or above.

### When should one start an unrelated donor search

Every AA patient lacking an HLA identical sibling, should activate a search for an unrelated donor at diagnosis, up to the age of 60 years. UD grafts have been reported to produce 90% survival, when given as first line therapy in children.^3^ However, older patients grafted from UD have inferior outcome, with 5 year survival of 85%, 77%, 66% and 49% for patients aged 1 to 10, 11 to 30, 30 to 40 and over 40 years of age.^4^ Therefore, ATG+CsA should be first line therapy in patients over 20 years of age, when an HLA identical donor is not available. In case of failure of IST, an UD graft would be the best option as second line therapy, for patients aged 20 to 60 years. Above the age of 60 SCT is associated with significant toxicity: in a recent analysis, mortality above the age of 60, was in the order of 50%.^2^

### Do we have a standard conditioning regimen?

Standard conditioning for matched sibling transplants under the age of 40 is Cyclophosphamide 200 mg/kg (CY 200) and ATG, as originally described. For older patients, current guidelines support the use of FLU-CY-ATG-low dose irradiation (FCA) or FLU-CY-alemtuzumab (CAMPATH) (FCC).^5▪^^,^^6▪^ The HLA matching between donor and recipient is relevant, and one should aim to identify an 8/8 HLA A,B,C,DRB1 matched unrelated donor. Rituximab 200 mg on day +5 should be added in patients receiving alternative donor grafts.^7^

### Bone marrow is the preferred stem cell source

The preferred stem cell source is unmanipulated bone marrow, as shown in registry based studies, with significant survival advantage of BM over GSCF mobilized PB.^8▪^^,^^9^ The reasons for the survival disadvantage of PB grafts can be summarized as follows: no reduction of primary or secondary graft failure (9% for both sources) and increased risk of acute and chronic GvHD (11% vs 22%).

### GvHD prophylaxis

**Figure 1:**
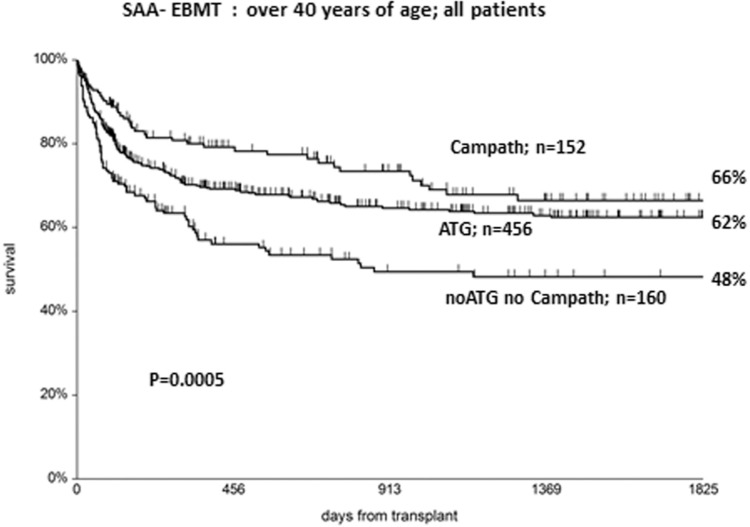
Actuarial survival of patients undergoing a SCT from HLA identical siblings or unrelated donors: shown is the effect of GvHD prophylaxis with Campath (alemtuzumab), ATG, or no Campath/ATG. The difference is highly significant.

Patients with AA have no benefit in developing acute and especially chronic GvHD, unlike patients with leukemia. For this reason, every effort must be made to reduce the risk at a minimum. We have already mentioned the preferred use of BM cells as a stem cell source. In addition, in vivo T cell depletion, with either ATG or Campath, should be considered for all AA patients, in combination with conventional CsA+methotrexate (MTX) prophylaxis. This will prevent GvHD and improve survival, as shown in Figure 1.

In a recent study,^10▪^ rabbit ATG was superior to horse ATG, in protecting patients from GvHD and improved survival in patients receiving UD grafts.

### Haploidentical donors and cord blood grafts

HLA haploidentical related donors (HAPLO) and unrelated cord blood (UCB) units are 2 additional options for patients who lack a matched sibling. HAPLO grafts are increasingly used and early results show survival in the range of 85% and a relatively low incidence of graft failure and GvHD with ATG based prophylaxis.^11^ High dose post-transplant CY (PT-CY) has also been used for HAPLO grafts in AA patients, with excellent early results.^12^ Unrelated cord blood is an additional option, when a matched donor is unavailable: a recent prospective trial of UCB grafts, with a cell dose equal or greater than 4 × 10^7^/kg, shows a 2-year survival of 84%.^7^

## Future perspective

### Conditioning regimen for older patients

The high mortality of patients over the age of 40, also with HLA matched donors, remains an issue.^2^ One option to be tested, would be the use of a reduced intensity regimen and optimal GvHD prophylaxis, such as the one proposed for HAPLO gtrafts^3^: this regimen includes ATG on days -8–7–6, then FLU CY and low dose TBI, followed by PTCY and CsA with mycophenolate.^12^ Reducing to a minimum GvHD may also reduce infections and toxicity.

### Rituximab

The recent UCB prospective trial^7^ has adopted the early administration on day +5 of rituximab, to prevent EBV related lymphoproliferative disorders. Rituximab day +5 is well tolerated, effective and may also reduce GvHD, and should be considered for all patients with AA undergoing an alternative donor transplant.

### Upfront alternative donor grafts

Neutropenia and early severe infections are an issue for patients with severe or very severe disease. First line transplants from UD has already been reported with success,^3^ and HAPLO donors are currently being tested in a prospective trial, following the encouraging results in Baltimore.^12^ More patients, especially adults, need to be treated before upfront alternative donor transplants can be considered outside a clinical trial.

### Treatment strategies

Trials are ongoing to confirm whether the triple combination ATG+CsA+ eltrombopag results in a 90% survival, when given as first line therapy in AA patients.^13▪^ Results of these trials will need to be considered when selecting the optimal treatment strategy for patients with AA, especially over the age of 40.
